# Impact of Harvest Time and Pruning Technique on Total CBD Concentration and Yield of Medicinal Cannabis

**DOI:** 10.3390/plants11010140

**Published:** 2022-01-05

**Authors:** Danilo Crispim Massuela, Jens Hartung, Sebastian Munz, Federico Erpenbach, Simone Graeff-Hönninger

**Affiliations:** 1 Cropping Systems and Modelling, Institute of Crop Science, University of Hohenheim, 70599 Stuttgart, Germany; s.munz@uni-hohenheim.de (S.M.); federico.erpenbach@uni-hohenheim.de (F.E.); simone.graeff@uni-hohenheim.de (S.G.-H.); 2 Biostatistics, Institute of Crop Science, University of Hohenheim, 70599 Stuttgart, Germany; jens.hartung@uni-hohenheim.de

**Keywords:** *Cannabis sativa*, cannabidiol, CBD yield, harvest time, pruning, topping

## Abstract

The definition of optimum harvest and pruning interventions are important factors varying inflorescence yield and cannabinoid composition. This study investigated the impact of (i) harvest time (HT) and (ii) pruning techniques (PT) on plant biomass accumulation, CBD and CBDA-concentrations and total CBD yield of a chemotype III medical cannabis genotype under indoor cultivation. The experiment consisted of four HTs between 5 and 11 weeks of flowering and three PTs-apical cut (T); removal of side shoots (L) and control (C), not pruned plants. Results showed that inflorescence dry weight increased continuously, while the total CBD concentration did not differ significantly over time. For the studied genotype, optimum harvest time defined by highest total CBD yield was found at 9 weeks of flowering. Total CBD-concentration of inflorescences in different fractions of the plant’s height was significantly higher in the top (9.9%) in comparison with mid (8.2%) and low (7.7%) fractions. The T plants produced significantly higher dry weight of inflorescences and leaves than L and C. Total CBD yield of inflorescences for PTs were significantly different among pruned groups, but do not differ from the control group. However, a trend for higher yields was observed (T > C > L).

## 1. Introduction

Worldwide cannabis cultivation has increased due to the recent changes in legalization, regulation and marketization of *Cannabis sativa* L. for industrial, medicinal and recreational use [1,2]. To date, 177 phytocannabinoids have been identified in cannabis plants [3], among which the two most abundant are the psychoactive compound Δ^9^-tetrahydrocannabinol (THC) and the non-psychoactive cannabidiol (CBD) [4]—whose medical properties have accumulated evidence for decades [5,6]. Both compounds have shown pharmacological effects for several medical treatments [7]. In the plant material, phytocannabinoids are plant secondary metabolites present in acidic forms, Δ^9^-tetrahydrocannabinolic acid (THCA) and cannabidiolic acid (CBDA) [8]. These are synthetized and deposited mostly on glandular trichomes of cannabis inflorescences, although also found in lower concentrations in other plant tissue, as leaves and roots [9,10].

The cultivation of Active Pharmaceutical Ingredients for medical applications demands standardization of product quality (i.e., cannabinoids, terpenes and flavonoids) and cultivation processes [11,12]. Therefore, medical cannabis is often cultivated in indoor and greenhouse systems enabling both more control of environmental conditions and thus a higher standardization of cultivation processes [12]. Since cannabis is a short-day plant, indoor systems also facilitate several growing cycles per year by controlling photoperiodism and temperature, especially important to enable year-round cultivation in sub-tropical and temperate regions [13]. The importance of optimizing indoor systems has increased due to the demand for yield maximization and improved efficiency of growing systems [13]. Final yield quantity and quality of inflorescences are highly variable and depends on numerous factors such as genotype [14,15,16]; the agronomic practices, such as irrigation and fertilizer regimes [17,18,19,20], light spectra [14,21], intensity [22,23] and photoperiod [24]; plant density [25]; environmental conditions (humidity and temperature) [12] and the influence of biotic and abiotic stresses [26,27]—including pruning and defoliation techniques [28,29]—and finally, the duration of the vegetative and generative period. Thus, so many differences can be found for reported yield of medicinal cannabis currently in literature.

A crucial question to maximize yields is the correct harvest time based on inflorescence maturity and biomass accumulation. The identification of the optimum harvest time for each genotype can assist in the optimization of cultivation processes as indoor cannabis is economically resource-intensive [30]. The typical duration of the generative period of conventional medical cannabis genotypes for indoor systems ranges from 7 to 14 weeks of flowering [31,32,33,34,35]. The composition of cannabinoids in inflorescences changes over time as cannabigerolic acid (CBGA) is synthesized in growing inflorescences, and both CBDA and THCA are converted from CBGA [14,15,16,17]. These conversions are determined by genomic expression of CBDA- and THCA-synthases, responsible for the content and ratios among cannabinoids in different chemotypes [36]. Chemotype III are CBD-dominant plants; in the experiment by [37], plants synthetized CBDA continuously until the end of the experiment with eleven weeks of flowering, while CBGA reached a maximum concentration around five weeks of flowering and decreased afterwards. In another study [16] also with chemotype III plants, genotypes presented maximum concentration of total CBD by six weeks of flowering, generally reaching a plateau with consequent reduction of concentrations after ten weeks of flowering. However, some genotypes already presented significant reduction of total CBD concentrations after seven weeks of flowering. In literature [31,38,39], it is suggested that the change in coloration of pistils and trichomes is due to flower maturity and plant senescence, and thus can indicate that the plant is ready to harvest. Cannabinoid and terpene metabolites are produced in glandular trichomes (bulbous, sessile and stalked) [38], that start to develop during the plant’s generative stage eventually covering the complete surface of inflorescences—composed by individual flowers, calyx, bracts, phytomeres and adjacent reduced leaves [39]. During this period, the trichomes “mature” and change coloration from transparent (I) to white (II) to amber/brown (III) as the ratio of compounds changes [4,37,40]. It is suggested that stage (I) is too early for harvest, as the plant continues to produce inflorescence matter, and this would reduce overall yields. The last stage (III) is also referred as being “too late”, since brown trichomes are linked with an advanced senescence stage of the plant and a potential loss of flower quality, with the conversion of THCA and CBDA into cannabinolic acid (CBNA) [31]. However, the continuous growth of inflorescences also leads to the continuous creation of lateral phytomeres, consequently leading to new trichome formation even in the later harvest events [39]. This can lead to biased observations on trichome color, which could result in misinterpretations for optimum harvest time definition. The ambiguity of stage transitions calls for the selection of appropriate sampling parameters and to define unbiased quantitative methods to correctly evaluate number and color of trichomes to indicate optimum harvest time—what is still missing in literature. Therefore, a time scale (weeks of flowering) was used and tested in this study as a possible definition of optimum harvest time.

Another important management factor is pruning, e.g., removing the apical meristem and/or branches and leaves, as this modulates plant architecture, plant biomass allocation and the yield of inflorescences and cannabinoids per plant and area [28,29]. The removal of the apical meristem changes hormone balances (e.g., auxin, cytokinin) in the plant stimulating the development of side shoots by the relieving of apical dominance [41], and thus, altering plant architecture, which can increase light penetration into the canopy and air circulation [24,25,26]. This can further lead to different micro-climates within the plant canopy due to inter-shoot shading [28,42] altering the concentration of cannabinoids in the inflorescences [29,40]. In a study by Folina et al., the effect of the apical cut (topping) in two hemp cultivars resulted in significantly higher total CBD content as pruning increased CBD concentrations [43]. Additionally, significantly higher inflorescence dry matter and leaf area were found for topped plants due to the high number of secondary shoots, while plant height, plant dry weight and number of nodes in the main shoots were significantly lower [43]. Recent publications [28,29] concluded that plant architecture modulation can increase the standardization and uniformity of cannabinoid concentration in the plant, therefore reducing inner-plant variability. This could be achieved by reducing the concentration gaps along the plant by increasing the cannabinoid concentration in bottom inflorescences. The researchers pointed out that the pruning techniques “single prune” (topping) and “1° branch removal” (i.e., removing lateral shoots at the main axis) visibly altered shoot structure and cannabinoid composition [28]. Therefore, the impact of pruning technique on the inner-plant uniformity of cannabinoid concentration needs to be considered.

Finally, as concluded in the review of medical cannabis indoor cultivation factors and practices by Jin et al. [44], due to a large number of variables, harvest time is subjective and not possible to be determined generally for cannabis plants, thus, the necessity to be examined on a case-by-case basis. Additionally, the available details on pruning techniques —including topping and the removal of side shoots—are limited. The authors suggested that methods need to be examined in controlled studies.

Therefore, the aim of this study was to investigate the impact of factors: (i) harvest times (HT) and (ii) different pruning techniques (PT) on inflorescence yield; CBD, CBDA and total CBD-concentrations and yield of a chemotype III medical cannabis genotype under indoor cultivation.

## 2. Materials and Methods

An indoor experiment was performed at the University of Hohenheim (Stuttgart, Germany) between August and December in 2020. The experiment was two-factorial with the treatment harvest time (HT) and pruning techniques (PT). The HT varied in four levels and PT varied in three levels. Treatments were arranged on horticultural tables according to a non-resolvable row-column design [45] with nine rows in four columns. All treatment combinations had three replicates.

Environmental conditions such as air temperature and humidity were monitored continuously within the cultivation room inside a greenhouse complex, built on double insulated glass and automated environmental regulation systems. The daily mean air temperatures varied from 19.2–32.2 °C, and relative humidity varied from 32.6–77.7% for the cultivation period. The average, minimum and maximum daily values are presented in Figure S1 in the Supplementary Materials.

During the vegetative period, the photoperiod was 18 h, provided by natural sunlight and supplemented by ceramic metal halide lamps, CHD Agro 400 W (DH Licht GmbH, Wülfrath, Germany). If solar radiation measured above the greenhouse was higher than 35 k Lux, supplemental lighting was turned off automatically. The total duration was 28 days.

During the generative period, black curtains with 95% obscuration performance were used to exclude solar radiation, and only the supplemental lighting was used and set to a photoperiod of 12 h.

### 2.1. Cultivation Methods

Experimental plants were generated by cloning standardized stock plants of a chemotype III genotype provided by Ai Fame GmbH (Wald-Schönengrund, Switzerland). Clones were generated by vegetative propagation from the apical tip of stock plants’ upper branches, dipped into Clonex^®^ (Growth Technology Ltd., Taunton Somerset, UK) and transferred into EazyPlugs (3.5 cm × 3.5 cm × 3.0 cm) (Goirle, The Netherlands). Clones were cultivated in a nursery greenhouse and were sprinkled and ventilated daily to guarantee humidity levels above 80% and proper air circulation. After 14 days, rooted clones were transplanted into 9 cm diameter round pots with 80% Klasmann Substrate-5 + Green Fiber (Klasmann-Deilmann GmbH, Geeste, Germany), mixed with 20% perlite of PerligranR Extra (KNAUF, Iphofen, Germany). The transplanting day is considered as the beginning of the experiment, being the first day after planting (DAP). After one week (7 DAP), the plants were transferred to square pots (15 cm × 15 cm × 20 cm) using the same substrate composition. An Arbuscular Mycorrhiza Fungi (AMF) mixture granulate “Mykorrhiza Granulat” (Tyroler Glueckspilze, Innsbruck, Austria) was added to the soil mixture during the first and second repot in a total amount of 3.76 g per pot. The pots were placed in four rows each with nine pots on horticultural tables (1.0 m × 2.5 m) with a density of 14.4 plants m^−2^. A drip irrigation system with controller was mounted in the pots to provide a constant water supply of 100–500 mL per day depending on the growing stage of the plants and environmental conditions.

For fertilization, the organic line of BioCanna (CANNA, North Brabant, The Netherlands) was used and applied three times a week. During the vegetative growth cycle, plants received root stimulator Biocanna Bio Rhizotonic (0.6-0.2-0.6) and organic fertilizer Biocanna Bio Vega (3.5-1.0-5.5). During the generative growth cycle, plants received the organic fertilizer Biocanna Bio Flores (2.5-2.2-5) and Biocanna Bio Boost (0.02-0.12-0.08). The dilution concentration over time followed the producer’s recommendation. Furthermore, elemental vitamins Hesi SuperVit (Hesi, The Netherlands) were added at every fertilizer solution with 1–2 drops/6 L. Additionally, foliar application of Emerald Shaman (Advanced Nutrients, Los Angeles, CA, USA) was applied three times a week (2 drops/L) from 0 DAP until 63 DAP by nebulizing it on the plants. More details about the fertilization scheme can be found in Table S2 in the Supplementary Materials.

Pests were controlled biologically through auxiliary predatory insect populations (spp. *Phytoseiulus persimilis*, *Amblyseius californicus*, *Orius majusculus* and *Aphidoletes aphidimyza*) provided weekly by the company Sautter & Stepper (Ammerbuch, Germany) against spider mites (*Tetranychus urticae*) and aphids (*Aphidoidea*). In addition, organic approved substances (Neem oil and Spruzit^®^) were applied in local spots for pest control.

#### 2.1.1. Harvest Time

The harvests were separated by two weeks and focused on the later stages of inflorescence maturation. The HTs used were 5, 7, 9 and 11 weeks of flowering. Harvest times were chosen based on a prior experiment conducted with the same genotype and HTs from 6 to 12 weeks of flowering.

#### 2.1.2. Pruning Techniques

Pruning was done by cutting the meristems and branches with disinfected clippers at 27 DAP. At this growth stage, the plants had between 10 and 14 internodes. The techniques applied were a control (C), representing the not pruned plants, topping (T)—the apical cut of the growing apex at the tenth node of the main stem and lollipop (L)—the removal of the two lowest side branches growing from the main shoot at 27 DAP, and in addition, the next two lowest branches at 36 DAP.

### 2.2. Sampling and Laboratory Analysis

**Figure 1:**
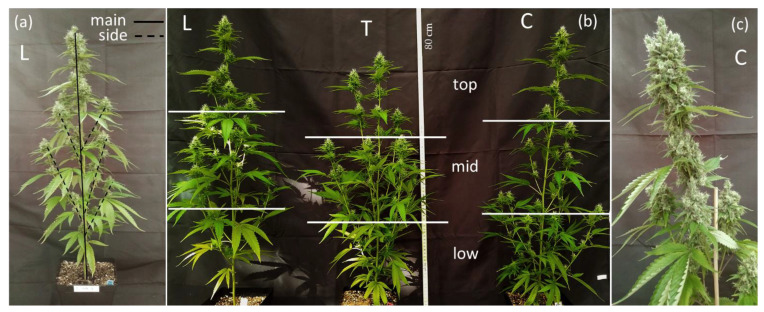
(**a**) Separation of main axis and side shoots in a lollipop (L) plant; (**b**) Plant architecture as modified by pruning techniques in plants at five weeks of flowering (65 DAP), shortly before the first harvest. Lollipop (L, left), topping at tenth node (T, center) and control (C, right). The horizontal lines show the separation of inflorescences into the three fractions (top, mid and low) based on each plant’s height; (**c**) Main top inflorescence of a control plant at nine weeks of flowering (93 DAP).

At each harvest, three plants per PT were cut at the base and separated into three fractions: stems, leaves and inflorescences. Each fraction was separated based on the location on the plant’s (i) main axis and (ii) side shoots (Figure 1a). In the case of T plants, the highest shoot from top, that held major dominance after apical pruning was considered as main axis. At the latter two harvests, separate samples were taken additionally from the top, middle and bottom (low) one-third of the plant (Figure 1b) to investigate inner-plant variation in total CBD concentration and yield. This factor is later referred to as inflorescence position.

The samples of stems and leaves were oven air-dried at 60 °C for 48 h. The inflorescences were immediately submersed in liquid nitrogen (−196 °C) to prevent further chemical reactions (i.e., oxidation and decarboxylation) and to maintain inflorescence quality. Samples were stored at −80 °C and later freeze-dried in the laboratory freeze-drier model P 15 K (−30 °C, +30 °C) (Dieter Pietkowski, Petershausen, Germany). The freeze-dried inflorescence samples were ground to a homogeneous powder. The residual moisture of each sample was measured with a moisture analyzer (DBS 60-3 of Kern and Sohn GmbH, Balingen, Germany).

The ground, freeze-dried samples were analyzed by high performance liquid chromatography (HPLC), which is the reference method for cannabinoid quantification. The HPLC analysis followed the method adapted by Burgel et al. [15]. The cannabinoid extraction was done using 100 ± 10 mg of freeze-dried inflorescences in 100 mL of a methanol 90%/chloroform, 10% (*v/v*) (9 + 1) composite in an ultrasonic bath for 30 min at 40 °C. After cooling down, the solution was filtered through syringe filters Polytetrafluorethylen (PTFE), 0.45 µm (Macherey-Nagel GmbH & Co. KG, Düren, Germany) into HPLC vials and injected into the HPLC system (1290 Infinity II LC System, Agilent, Santa Clara, CA, USA) equipped with an autosampler, a quaternary pump, as well as a diode-array spectrophotometer (DAD) at the detection wavelength of 230 nm. The chromatographic separation was carried out on a Nucleosil 120-3 C8 column (125 mm × 4 mm i.d., 3.0 µm) with a guard column EC 4/3 Nucleosil 120-3 C8 (Macherey-Nagel, Oensingen, Switzerland). The mobile phase was a mixture of HPLC-grade methanol (solvent A) and 0.1% acetic acid in HPLC-grade distilled H_2_O (solvent B; Sigma-Aldrich, Saint Louis, MO, USA) at a constant flow rate of 0.7 mL min^−1^ with gradient elution mode. The injection volume was 10 µL and the total run time comprised 27 min. The integration of targeted peaks was done using cannabinoids analytical reference standards for CBD (C-045) and CBDA (C-144) (Sigma-Aldrich, Darmstadt, Germany) and data analysis was carried out with the software ChemStation for LC Rev. B.04.03-SP2 (Agilent, Santa Clara, CA, USA). Calibration curves were created from diluted standard solutions with a coefficient of determination of 1.0 for both CBD and CBDA. The limit of detection for CBD and CBDA was 0.0015%.

### 2.3. Calculations

Total CBD concentration (%) was calculated as a weighted sum of CBD (%) and CBDA (%) in each inflorescence sample. The multiplication by the factor 0.877 accounts for the differences in molar mass between the acid and neutral forms of the cannabinoid, as one molecule of CO_2_ is lost during decarboxylation:

(1) $${\mathrm{CBD}}_{\mathrm{Total}}=\mathrm{CBD}+\mathrm{CBDA}\times 0.877$$

To correctly evaluate cannabinoid production capacity, the calculated yield of total CBD (mg·plant^−1^) must be taken into consideration to properly evaluate the factors of HT and PT. Yield was calculated considering inflorescence fresh weight, the residual moisture of the analytical sample and the total CBD concentration, using Equation (2). The conversion factor 0.2 represents the average dry matter concentration of fresh inflorescences and was applied to calculate the yield at the moisture of the analytical sample. The residual moisture was the weight proportion of water in the analytical freeze-dried samples (ranged from 0.027–0.063). The total CBD yield was calculated for each sample as follows:

(2) $${\mathrm{CBDyield}}_{\mathrm{Total}}{=\mathrm{CBD}}_{\mathrm{Total}}\;\cdot \mathrm{Inflorescence}\;\mathrm{fresh}\;\mathrm{weight}\times (0{.2\;\times (1\;-\;\mathrm{residual}\;\mathrm{moisture})}^{-1})$$

### 2.4. Statistical Analysis

The experiment was analyzed using a mixed model approach for all traits, which were determined by the measurement of single plants. The model can be described as:

(3) $$y_{\mathrm{ijkl}}{=\mu +\alpha }_{i\;}{+\beta }_{j\;}+{\left(\alpha \beta \right)}_{\mathrm{ij}}+r_{k}+c_{l}{+e}_{\mathrm{ijkl}},$$

where $y_{ijkl}$ is the *kl*th observation of pruning technique *i* at harvest *j*, μ is the intercept, $\alpha_{i}$, $\beta_{j}$ and ${\left(\alpha \beta \right)}_{ij}$ are the fixed main effects for pruning technique *i*, harvest *j* and its interaction effects, $r_{k}$ and $c_{l}$ are the random row and column effects of the *k*th row and *l* th column from the design, respectively, and $e_{ijkl}$ is the confounded effect of plant and error corresponding to $y_{ijkl}$. The model was allowed to fit heterogeneous error variances and thus, pruning technique-specific, harvest time-specific and pruning technique-by-harvest time-specific error variances and the best model was selected via AIC [46]. Note that pruning techniques vary in their variance for most traits, but the best model always fits harvest-specific error variances. Further note that variances for design effects were generally small compared to error variances and often bounded at zero. Thus, if convergence problems occurred when fitting heterogeneous variances, design effects were dropped from the model to get convergence. Further note that harvest was measured in weeks and thus can be modeled as metric as well. This would allow to fit linear and quadratic trends, but in our case, the lack of fit test remains significant even for a quadratic polynomial. Thus, harvest was treated as factor. In case of finding significant differences via global F test, a Fisher’s LSD test with α = 0.05 was used for pairwise comparison and a letter display was derived after [47].

The effect of inflorescence position in plants’ fractions (top, mid, low) and pruning technique at optimal harvest (HT = 9weeks of flowering) were analyzed with a mixed model analogous to (3) but replacing harvest time by fraction. Additionally, a first-order autoregressive variance-covariance structure with homogeneous or heterogeneous fraction-specific variances were fitted to account for repeated measures per plant. Again, the best model was selected via AIC. All statistical analyses were conducted by using the software SAS version 9.4 (The SAS Institute, Cary, NC, USA).

## 3. Results and Discussion

The interactions between the factors of HT and PT was not significant for plant biomass, total CBD concentration and total CBD yield. Therefore, results are presented separately for HT and PT. Results of the analysis of variance for all target variables can be found in Figure S1, in the Supplementary Materials.

### 3.1. Effect of Harvest Time (HT)

Inflorescences (Figure 1c) started to appear after the second week of the generative phase, when growth of plant height stagnated, after 58 DAP, and continued to grow steadily until the end of the experiment.

#### 3.1.1. Biomass Accumulation by Harvest Time

**Figure 2:**
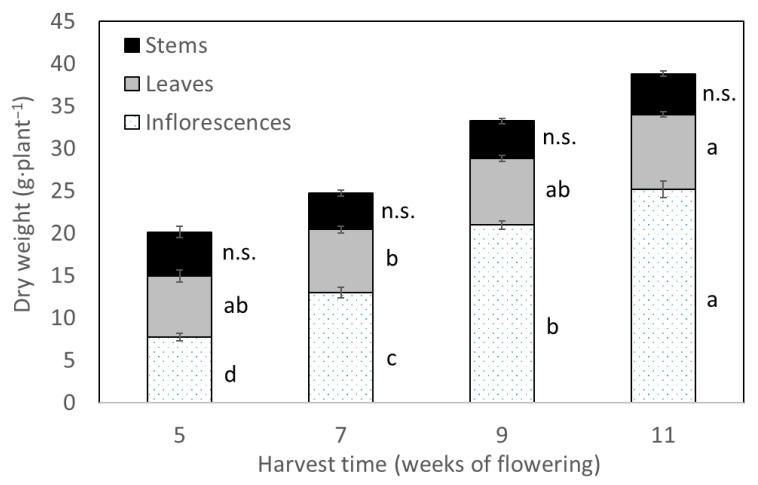
Least square means (±standard error) of dry weights (g·plant^−1^) of stems, leaves and inflorescences for each of the four harvest times (weeks of flowering). For each plant organ, means with at least one identical letter are not significantly different from each other according to Fisher´s LSD test with α = 0.05. Values for stems are not significantly (n.s.) different from each other according to global F-test.

During the generative phase, the total dry weight of plants increased continuously until final harvest of the experiment. The factor HT was statistically significant for biomass of inflorescences and leaves; the average dry weight of inflorescences per plant increased from 7.7 g at five weeks of flowering to a maximum of 25.1 g at eleven weeks of flowering (Figure 2). The highest biomass accumulation of leaves per plant was 8.8 g at the final HT.

#### 3.1.2. Cannabinoid Concentration and Yield by Harvest Time

Cannabinoid concentration was measured for CBD and CBDA with total CBD calculated using Equation (1).

**Figure 3:**
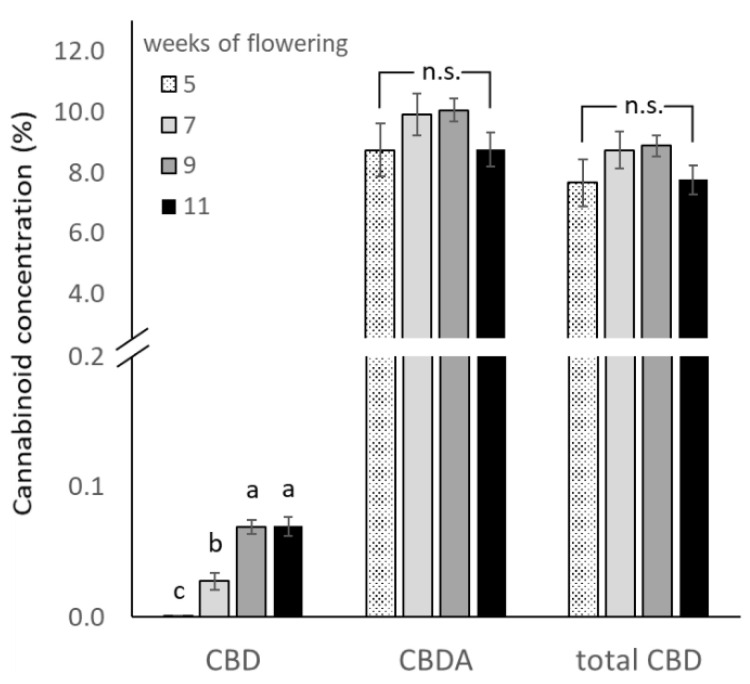
Least square means (±standard error) of CBD, CBDA and total CBD concentration (in % of inflorescence dry weight) for the four harvest times (weeks of flowering). For CBD values, means with at least one identical letter are not significantly different from each other according to Fisher´s LSD test with α = 0.05. Values for CBDA and total CBD are not significantly (n.s.) different from each other according to global F-test.

Total CBD concentration increased from 7.62% to a maximum of 8.88% by the third HT and decreased to 7.76% in the last HT (Figure 3). For CBDA and total CBD, differences among HT levels were not significant, although a trend of increasing and later decreasing concentrations can be observed. Such a temporal trend of cannabinoid concentrations in inflorescences was also reported by other authors for chemotype III [16,40,48] and chemotype I [35,37] cannabis plants. The reduction in concentration of cannabinoids can be related to plant senescence reducing cannabinoid synthesis [16]. Plants might have a maximum cannabinoid production capacity [49], so lower concentrations on later harvest may be due to dilution effects of cannabinoid contents in relation to inflorescence biomass. The significant increase of CBD concentrations in the later HTs infers that time increases exposure of phytocannabinoids to oxidation and decarboxylation processes in the trichomes [50,51]. On the other hand, sample preparation was successful in maintaining phytocannabinoids in their acidic form, as naturally synthetized by the plant. Therefore, the values of CBD are much smaller than CBDA values.

In the study from Yang et al. [17], similar trends for a plateau of total CBD concentrations in the studied range of harvest times were reported for five CBD-rich cannabis genotypes grown in open-field. All five genotypes presented an increasing trend of total CBD concentration until a peak by six to seven weeks of flowering. Two genotypes presented a plateau of concentration until ten weeks post anthesis, while in three genotypes, total CBD concentrations declined after the peak as plants aged. The concentrations varied from 4% to 12% in the cultivated genotypes [17]. The results for total CBD are also comparable to results found by Burgel et al. [42] in a similar indoor cultivation system also with chemotype III plants, with total CBD ranges of 5.97% to 6.22%.

Finally, the factor HT was significant for total CBD yield per plant. There was a steady increase of CBD yield starting from 415.0 mg·plant^−1^ at the first HT, while reaching a maximum value of 1334.9 mg·plant^−1^ at the last HT, not varying significantly to the third HT (Table 1). The last HT yielded the highest inflorescence dry weight and yield (Figure 2). However, this did not increase the capacity of the plant to synthetize more CBDA or CBD, suggesting a maximum capacity of the inflorescences to continuing producing cannabinoids as a possible limitation of assimilates’ production, and water, nutrient and light absorption [40]. This can be related to senescence processes related to plant aging [17]. Additionally, the secretion of cannabinoids in leaves’ tissue can cause necrosis and cell death via mitochondrial dysfunction [43].

Values for total CBD yield suggested that the optimum time for harvest of the tested genotype under the given conditions is around nine weeks of flowering, since CBD yield did not increase significantly in the last HT and this time would rather be invested in the vegetative period, promoting bigger plants with the same cultivation duration. For reference, other authors found similar results for the duration of the generative phase for medical cannabis. In a survey from commercial suppliers of 200 chemotype I and II cannabis genotypes available in Europe in 2011, 88% of the varieties had a recommended flowering period of seven to nine weeks [51]. This recommendation also falls in the range of commercially cultivated genotypes for the production of the EU registered pharmaceutical Sativex^®^ (GW Pharmaceuticals, UK) [52]. However, there is few reporting of optimum harvest time based on total CBD yield in literature for chemotype III varieties [16,37].

### 3.2. Effect of Pruning Technique (PT)

Cannabis plant development is monopodial with a continuous phytomere production regulated by apical dominance [39]. The natural growth behavior of the tested CBD-rich genotype (when not pruned) is to expand the main stem and side shoots, forming the characteristically cannabis triangular “Christmas tree” shape and accumulating biomass and inflorescences across the entire plant height (Figure 1b). The largest terminal inflorescence is found at the main apex (Figure 1c). The pruning techniques modified plant architecture as exemplarily illustrated in Figure 1b. On one hand, the apical cut treatment (T) reduced the height of the plants in comparison to the control group, but promoted the development and elongation of side shoots, generating several terminal inflorescences. On the other hand, L plants grew taller than the control plants, but with a lower number of side shoots than C and T plants (data not shown). The increase in plant’s height is possibly due to a higher deposition of cytokinin to the apical meristem, inducing enhanced meristem activity and plant elongation [28].

#### 3.2.1. Biomass Allocation by PT

The biomass allocation by plant organ (stems, leaves and inflorescences) and location (main axis or side shoots) are presented for all PTs in Table 2. Topped plants (T) produced significantly more leaf and inflorescence biomass compared to C and L. The removal of apical dominance with pruning leads to a redirection of hormones (e.g., auxins) and assimilates of the plant to the side shoots [53]. This way, T plants formed longer bottom shoots with significantly larger total biomass of stems, leaf and inflorescences than C plants. These differences were mainly due to larger biomasses at side shoots, whereas no significant differences were found for the main axis. T had, in general, three longer branches with terminal inflorescences, with a higher spatial distribution of the shoots and inflorescences per area (data not shown), forming a “rhombus” shape. Similar architecture modulation were also found for drug-type cannabis [29] and for fiber-type cannabis genotypes [54]. In both references, topping generated a higher number of side shoots, thus more top terminal inflorescence biomass than the control plants (not pruned).

Due to removal of lateral shoots, L plants had significantly lower side leaves’ biomass than other treatments. The lower amount of leaves in L plants could finally lead to the lower inflorescence’s biomass production in comparison to the topped plants, as the plants would possess lower leaf area and capacity to produce assimilates. Interestingly, L plants do not differ significantly to control plants for any category of biomass accumulation presented besides side leaves.

As cited in the introduction section, the source of variation for biomass accumulation and inflorescence yield are manifold and continually being reported in the literature. Factors as genotype, pot size, fertilization scheme, plant density, light intensity, indoor growing conditions, the duration of flowering period and management practices were reviewed by Jin et al. [44] and Backer et al. [55]. Authors cite that pruning can enhance yield by maximizing light interception, optimizing nutrient allocation and by creating more air circulation [44]. Calculated inflorescence yields per area for each PT-L (226.1 g·m^−2^), C (234.7 g·m^−2^) and T (266.4 g·m^−2^)—are comparable to results found by Knight et al. [56] (274.8 g·m^−2^) and yields reported in other studies [51].

#### 3.2.2. Cannabinoid Concentration and Total CBD Yield by PT

The total CBD concentration was not significantly affected by the pruning techniques (data not shown). The significantly higher biomass of inflorescences and leaves from T plants (Table 2) did not result in significantly higher total CBD yield (Table 3). Particularly, higher total CBD yields could be observed at the defined optimum HT (9 weeks of flowering) for T (1431.6 mg·plant^−^^1^) in comparison to C (1234.3 mg·plant^−^^1^) and L (1133.9 mg·plant^−^^1^). However, these differences were not significant although a trend following T > C > L was indicated (*p*-value 0.0923).

The lower total CBD yield of L plants can be related to the lower biomass of leaves and inflorescences. It was presumed that due to lateral shoot removal, L plants had less leaves for the production of assimilates and metabolites, thus impacting overall CBD production. In the studies of Danziger and Bernstein [28,29], the excessive removal of branches in one of the treatments caused lower available-photosynthetically leaf area and restricted energy availability in the plants, reducing the inflorescence yield and cannabinoid content of the plants.

#### 3.2.3. Inflorescence Position by PT—Inner Plant Variability

As seen in Figure 1b, each PT displayed unique patterns in biomass allocation along the plant height. The biomass allocation of inflorescence fresh weight by inflorescence position—top, mid and low—is presented in Table 4.

Across pruning techniques, control plants accumulated the lowest inflorescence biomass in the top fraction (23.5 g·plant^−1^) and the highest biomass of inflorescences in the low fraction (13.6 g·plant^−1^). In comparison to C, T plants showed a shift of inflorescence biomass from the low (10.4 g·plant^−1^) to the mid (35.9 g·plant^−1^) fraction due to the elongation of lower side shoots. This also resulted in 19% more inflorescence biomass in the top fraction compared with C, although not being significantly different. Furthermore, L plants presented significantly higher biomass in the top main inflorescence (33.9 g·plant^−^^1^) compared to C while reducing the weight in the low (6.7 g·plant^−^^1^) and mid (26.7 g·plant^−^^1^) fractions.

For the total CBD concentration, the inflorescence position showed significant differences for the top fraction (9.9%), which was significantly higher than mid (8.2%) and low (7.4%) fractions (Table 5). The significantly higher CBD-concentration in the top section is confirmed by another study in not pruned plants [40]. The authors reported decreasing cannabinoid concentrations at lower inflorescence position, as an effect from shading by higher branches.

The factor PT was not significant for total CBD concentrations. However, the yield of total CBD was significantly influenced by both factors, PT and inflorescence position. Inflorescences in the low fractions (156.8 mg·plant^−^^1^) had a lower total CBD yield than mid (533.2 mg·plant^−^^1^) and top (576.5 mg·plant^−^^1^) fractions. When analyzing PT considering the influence of inflorescence position, treatment L showed the lowest average total CBD yield (377.9 mg·plant^−^^1^), which was significantly different from the highest values found for T, yielding an average of 477.2 mg·plant^−^^1^. However, no significant differences were found in comparison to C plants (411.5 mg·plant^−^^1^).

The results showed the variability of total CBD-concentration and -yield between plants of each PT, as well as the inner plant variability when observing inflorescence position. In the experiments by Danziger and Bernstein [28,29] no significant differences were found for plant fresh biomass, inflorescence yield, CBDA and THCA concentrations between control and the single prune (topping) treatment. However, plant architecture modulation via pruning increased the cannabinoids’ uniformity along the plant by increasing the cannabinoid concentration in lower fractions. The researchers hypothesized that the modulation of plant architecture affects the microclimate in the plant shoot, impacting cannabinoid production at the inflorescence level [28]. The researchers also highlighted the importance of defining clear guidelines and regulation mechanisms of chemical variability within inflorescences as a tool for further developing plant architecture modulation techniques to optimize standardization of industrial cultivation [29]. In our study, the observed significantly higher total CBD concentrations in the top fractions are not confirmed for total CBD yield, mainly due to a higher biomass of inflorescences in the mid-fraction. Although CBD concentration was not significant for PT, the results of architecture modulation altering the inflorescences’ fresh weight distribution (Table 4) did generate significant differences for CBD yield, following the trend T > C > L. Furthermore, a higher number of replicates per treatment is necessary to better estimate the variability caused by pruning interventions.

Finally, the most suitable pruning technique ultimately depends on the cultivation objectives and the industrialization processes applied to the cultivation system. Management intensity may be reduced with unpruned plants; however, cultivators need to keep in mind the accumulation of inflorescences in the lower fractions, which contain lower levels of total CBD concentration and yield. Treatment L showed the lowest CBD yields; however, it presented an interesting compact architecture of a single main top terminal inflorescence with increased height, which may favor higher density systems like the SOG (sea of green) and the automatization of harvest process (e.g., using electric tumbler blade trimmers). In this case, a lower yield per plant can be overcome by a higher yield per area. The treatment T can be interesting to limit the number of developed side shoots of a plant and increase the number of inflorescences in the top fractions, thus shaping a desired plant architecture and a more even canopy distribution. For economic reasons, the objectives often are to maximize CBD yields in indoor systems with the shortest duration of the generative cycle and fewer inputs (time, labor, water, energy). In this case, the plant top fractions—which contains the highest total CBD concentration and content—would be the most interesting final product delivered as dry inflorescences, which often require labor- intensive manual harvest and trimming of inflorescences. For process optimization, the other fractions (mid and low) could be harvested for the production of extracts and concentrates with a higher mechanization level—but which can lead to higher harvest losses and the reduction of inflorescences’ size and quality when the final product’s object is medical dry inflorescences.

## 4. Conclusions

This study showed that the optimum time of harvest of the tested CBD-rich genotype was around nine weeks of flowering. The experimental factor pruning technique (PT) was efficient in altering plant architecture and biomass allocation, with significantly higher inflorescences’ dry weight in T plants, but which presented no advantages in producing significantly higher CBD yields, although a trend T > C > L was observed. Furthermore, the results on inner plant variability indicated significantly higher total CBD concentrations in the top fraction and significantly lower total CBD yields in the low fraction of the plant. This can indicate an advantage of topping plants, thus shifting the plant biomass from low to mid and top fractions in comparison with control plants. When accounting for the differences of inflorescence position, producers need to be aware of the total CBD concentration variability and should evaluate different PTs to fulfill their production goals and to optimize cultivation systems. Future work on yield optimization should consider the significance of inflorescence position and the impacts of plant architecture modulation.

## Figures and Tables

**Table 1:** Least square means (±standard error) of cannabinoid concentration (%) and total CBD yield (mg·plant^−1^) for the four harvest times (weeks of flowering). Means with at least one identical letter are not significantly different from each other according to Fisher´s LSD test with α = 0.05. Values for CBDA and total CBD concentration are not significantly different from each other according to global F-test. Note that *p*-values correspond to F-test within an ANOVA.

Harvest Time (Weeks of Flowering)	Cannabinoid Concentration (%)			Total CBD Yield (mg Plant^−1^)
	CBD	CBDA	Total CBD	
5	0 ± 0 c	8.73 ± 0.87	7.66 ± 0.77	415.0 ± 52.9 c
7	0.03 ± 0.01 b	9.91 ± 0.68	8.72 ± 0.61	785.4 ± 69.2 b
9	0.07 ± 0.01 a	9.87 ± 0.41	8.73 ± 0.37	1266.6 ± 41.6 a
11	0.07 ± 0.01 a	8.84 ± 0.54	7.84 ± 0.48	1334.9 ± 127.3 a
**Source of variation**	***p*-values**			
Harvest Time (HT)	<0.0001	0.2703	0.2767	<0.0001
Pruning Technique (PT)	0.1142	0.3026	0.2972	0.0923
HT × PT interactions	0.5955	0.7769	0.7842	0.6811

**Table 2:** Least square means (±standard error) of dry matter (g·plant^−1^) in different plant organs (stem, leaves and inflorescences) and locations (total, main axis and side shoots) for each pruning technique (PT). For each plant organ and location, means with at least one identical letter are not significantly different from each other according to Fisher´s LSD test with α = 0.05. Pruning techniques (C—control; L—lollipop; T—topping). Note that *p*-values correspond to F-test within an ANOVA.

Plant Organ	Location	Dry Matter (g·Plant^−1^)			*p*-Values for PT
		PT			
		C	L	T	
Stems	Total	4.3 ± 0.4 a	4.4 ± 0.4 a	5.5 ± 0.4 a	0.1005
	Main axis	2.8 ± 0.3 a	3.1 ± 0.3 a	2.4 ± 0.3 a	0.3853
	Side shoots	1.6 ± 0.2 b	1.3 ± 0.2 b	2.9 ± 0.2 a	<0.0001
Leaves	Total	7.6 ± 0.4 b	6.9 ± 0.4 b	9 ± 0.4 a	0.0052
	Main axis	2.9 ± 0.1 b	3.6 ± 0.1 a	2.6 ± 0.1 b	0.0013
	Side shoots	4.7 ± 0.3 b	3.2 ± 0.3 c	6.4 ± 0.3 a	<0.0001
Inflorescences	Total	16.3 ± 0.6 b	15.7 ± 0.6 b	18.5 ± 0.6 a	0.0117
	Main axis	4.8 ± 0.4 a	5.4 ± 0.4 a	4.8 ± 0.4 a	0.4226
	Side shoots	11.5 ± 0.6 b	10.2 ± 0.6 b	13.7 ± 0.6 a	0.0036

**Table 3:** Least square means (±standard error) of total CBD yield (mg·plant^−1^) for the different harvest times (weeks of flowering) by each pruning technique (PT). Means of HT with at least one identical letter are not significantly different from each other according to Fisher´s LSD test with α = 0.05. Values for PT are not significantly different from each other according to global F-test. Pruning techniques (C—control; L—lollipop; T—topping). Note that *p*-values correspond to F-test within an ANOVA.

Harvest Time (Weeks of Flowering)	Total CBD Yield (mg·Plant^−1^)			
	Means of Harvest Time	PT		
		C	L	T
5	415.0 ± 52.9 c	517 ± 91.6	313.2 ± 91.6	414.7 ± 91.6
7	785.4 ± 69.2 b	835.5 ± 119.9	718 ± 119.9	802.6 ± 119.9
9	1266.6 ± 41.6 a	1234.3 ± 72	1133.9 ± 72	1431.6 ± 72
11	1334.9 ± 127.3 a	1365.3 ± 220.4	1111.4 ± 220.4	1528.1 ± 220.4
**Source of variation**	***p*-values**			
Harvest Time (HT)	<0.0001			
Pruning Technique (PT)	0.0923			
HT × PT interactions	0.6811			

**Table 4:** Least square means (±standard error) of inflorescence fresh weight (g·plant^−1^) and weight fraction for each pruning technique (PT) for the third harvest time (9 weeks of flowering). Means for each PT (lowercase letters, in columns) and for each inflorescence position (uppercase letters, in rows) with at least one identical letter are not significantly different from each other according to Fisher´s LSD test with α = 0.05. Pruning techniques (C—control; L—lollipop; T—topping). Note that *p*-values correspond to F-test within an ANOVA.

Inflorescence Position	Inflorescence Fresh Weight (g·Plant^−1^)		
	PT		
	C	L	T
Top	23.5 ± 2.4 a B	33.9 ± 2.4 a	27.9 ± 2.4 a AB
Mid	30.7 ± 2.4 a AB	26.7 ± 2.4 a B	35.9 ± 2.4 a
Low	13.6 ± 2.4 b A	6.7 ± 2.4 b A	10.4 ± 2.4 b A
**Source of variation**	***p*-values**		
Pruning Technique (PT)	<0.0001		
Inflorescence position	0.2243		
PT × Inflorescence position interactions	0.0215		

**Table 5:** Least square means (±standard error) of total CBD concentration (%) and total CBD yield (mg·plant^−1^) for each pruning technique (PT) and inflorescence position at the third harvest time (9 weeks of flowering). Means for each PT and for each inflorescence position with at least one identical letter are not significantly different from each other according to Fisher´s LSD test with α = 0.05. Means for PT for total CBD (%) are not significantly different from each other for PT according to global F-test. Pruning techniques (C—control; L—lollipop; T—topping). Note that *p*-values correspond to F-test within an ANOVA.

Pruning Technique	Total CBD (%)	Total CBD Yield (mg Plant^−1^)
C	8.5 ± 0.6	411.5 ± 24.6 ab
L	7.9 ± 0.6	377.9 ± 24.6 b
T	9.0 ± 0.6	477.2 ± 24.6 a
**Inflorescence position**		
Top	9.9 ± 0.5 a	576.5 ± 27.4 a
Mid	8.2 ± 0.5 b	533.2 ± 27.4 a
Low	7.4 ± 0.5 b	156.8 ± 27.4 b
**Source of variation**	***p*-values**	
Pruning Technique (PT)	0.5207	0.0500
Inflorescence position	0.0028	<0.0001
PT × Inflorescence position interactions	0.7341	0.0597

## Data Availability

Not applicable.

## Supplementary Materials

The following are available online at https://www.mdpi.com/article/10.3390/plants11010140/s1, Figure S1: Histogram with environment data (air temperature and relative humidity) during the cultivation period, Table S1: Summary of *p*-values for the analyzed traits for factors of HT, PT and the interactions HT*PT, Table S2: Fertilization scheme.

## Abbreviations

HT	Harvest Time
PT	Pruning technique
C	Control
T	Topping
L	Lollipop
DAP	Days after planting
THC	Δ9-Tetrahydrocannabinol
CBD	Cannabidiol
CBDA	Cannabidiolic acid
THCA	Δ^9^-Tetrahydrocannabinolic acid
CBGA	Cannabigerolic acid
CBNA	Cannabinolic acid
