# Supplementary material for: Impact of Harvest Time and Pruning Technique on Total CBD Concentration and Yield of Medicinal Cannabis
Source: Plants (Basel). 2022 Jan 5;11(1):140. doi: 10.3390/plants11010140 (PMC8747189; doi:10.3390/plants11010140)
Supplement: Supplementary file 1 [file plants-11-00140-s001.zip › plants-1400037-supplementary.pdf]

plants

MDPI

Supplementary material

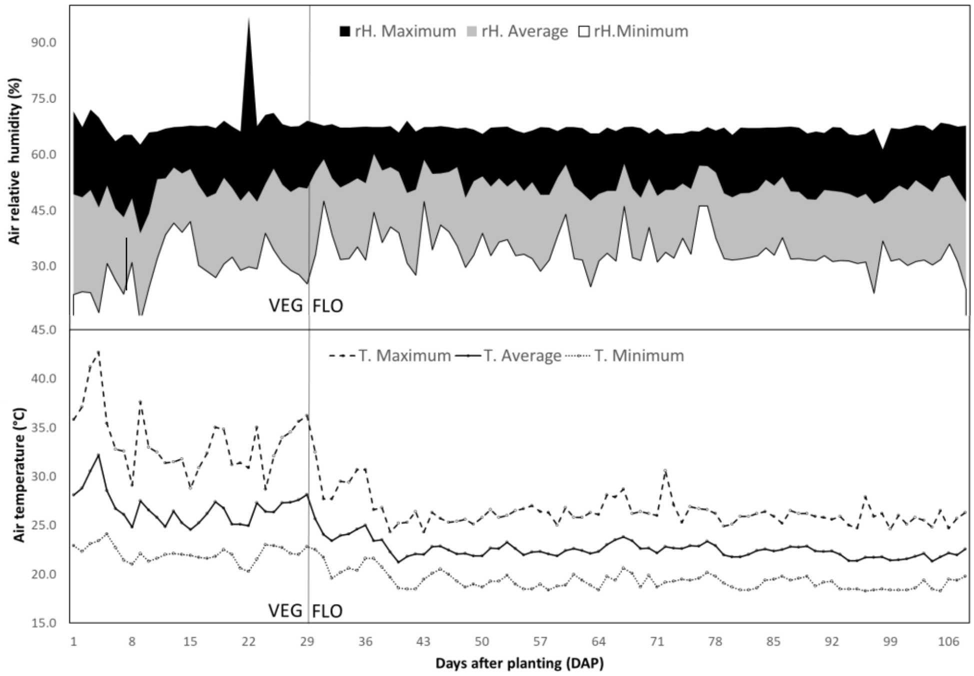

Figure S1. Histogram with environment data collected by greenhouse sensors during the cultivation period, represented in x axis as days after planting (DAP). The upper graph plots daily averages mean, maximum and minimum air relative humidity (rH %). The lower graph plots daily averages mean, maximum and minimum air temperature (°C). The vertical bar indicates the correspondent vegetative period (VEG) and the generative period (FLO), the shift to a short-day photoperiod happened at DAP 29.

Table S1. Summary of p-values for the analyzed traits for factors HT, PT and the interactions HT*PT.

	Factors		
p values for analyzed traits	HT	PT	HT*PT
Inflorescence fresh weight (mg.plant^{-1})	<.0001	0.2243	0.0215
CBDA %	0.2703	0.3026	0.7769
CBD %	<.0001	0.1142	0.5955
total CBD %	0.2767	0.2972	0.7842
total CBD Yield (mg.plant^{-1})	<.0001	0.0923	0.6811
Inflorescences dry weight (g.plant^{-1})	-	0.0117	-
Leaves dry weight (g.plant^{-1})	-	0.0052	-
Stems dry weight (g.plant^{-1})	-	0.1005	-

Table S2. Fertilization scheme. The total amount of products (ml) per plant is presented for each organic fertilizer (R – BioCanna Rhizotonic, V - BioCanna Vega, F - BioCanna Flores, B - BioCanna Boost) used during the entire cultivation period (VEG+FLO). Additionally, the amount of fertilizer provided in each harvest time is declared together with the amounts for VEG (vegetative period) and FLO (generative period) solely.

			Amounts (ml of product per plant)				Total fertilizer	VEG fertilizer	FLO fertilizer
**Experiment**	**HT**	**DAP**	**R**	**V**	**F**	**B**	**(ml plant $^{-1}$)**	**R + V**	**F + B**
**Total**			7.8	4.5	26.9	20.0	59.2	12.3	46.9
	1	65			8.9	9.3			18.2
	2	79			16.2	15.5			31.6
	3	93			24.3	20.0			44.3
	4	107			26.9	20.0			46.9
